# Comparative Analysis of Glycoside Hydrolases Activities from Phylogenetically Diverse Marine Bacteria of the Genus *Arenibacter*

**DOI:** 10.3390/md11061977

**Published:** 2013-06-10

**Authors:** Irina Bakunina, Olga Nedashkovskaya, Larissa Balabanova, Tatyana Zvyagintseva, Valery Rasskasov, Valery Mikhailov

**Affiliations:** 1 Laboratory of Enzyme Chemistry, Laboratory of Microbiology and Laboratory of Molecular Biology of G.B. Elyakov Pacific Institute of Bioorganic Chemistry, Far Eastern Branch, Russian Academy of Sciences, Vladivostok 690022, Russia; E-Mails: olganedashkovska@yahoo.com (O.N.); lbalabanova@mail.ru (L.B.); zvyag@piboc.dvo.ru (T.Z.); raskaz@piboc.dvo.ru (V.R.); mikhailov@piboc.dvo.ru (V.M.); 2 School of Natural Sciences, Far Eastern Federal University, Vladivostok 690091, Russia

**Keywords:** *Bacteroidetes*, *Arenibacter*, glycoside hydrolase, α-*N*-atcetylgalactosaminidase, β-*N*-acetylglucosaminidase

## Abstract

A total of 16 marine strains belonging to the genus *Arenibacter*, recovered from diverse microbial communities associated with various marine habitats and collected from different locations, were evaluated in degradation of natural polysaccharides and chromogenic glycosides. Most strains were affiliated with five recognized species, and some presented three new species within the genus *Arenibacter*. No strains contained enzymes depolymerizing polysaccharides, but synthesized a wide spectrum of glycosidases. Highly active β-*N*-acetylglucosaminidases and α-*N*-acetylgalactosaminidases were the main glycosidases for all *Arenibacter*. The genes, encoding two new members of glycoside hydrolyses (GH) families, 20 and 109, were isolated and characterized from the genomes of *Arenibacter latericius*. Molecular genetic analysis using glycosidase-specific primers shows the absence of GH27 and GH36 genes. A sequence comparison with functionally-characterized GH20 and GH109 enzymes shows that both sequences are closest to the enzymes of chitinolytic bacteria *Vibrio furnissii* and *Cellulomonas fimi* of marine and terrestrial origin, as well as human pathogen *Elisabethkingia meningoseptica* and simbionts *Akkermansia muciniphila*, gut and non-gut *Bacteroides*, respectively. These results revealed that the genus *Arenibacter* is a highly taxonomic diverse group of microorganisms, which can participate in degradation of natural polymers in marine environments depending on their niche and habitat adaptations. They are new prospective candidates for biotechnological applications due to their production of unique glycosidases.

## 1. Introduction

*Bacteroidetes* represents one of the main evolutionary branches of the domain *Bacteria* and one of the dominant components of the marine microbiota. Due to multienzyme complexes facilitating disposal of any natural substrates as sources of carbon and energy, marine *Bacteroidetes* are considered to be a promising source of unique, biologically active compounds.

The genus *Arenibacter*, a member of marine clade of the family *Flavobacteriaceae* of the phylum *Bacteroidetes*, was proposed by Ivanova *et al.* [1] to accommodate Gram-negative, heterotrophic, aerobic and pigmented bacteria of marine origin. At the time of writing, the genus *Arenibacter* comprises six recognized species: Arenibacter certesii, *Arenibacter* *echinorum*, Arenibacter latericius, *Arenibacter* *nanhaiticus*, Arenibacter troitsensis, *Arenibacter* *palladensis* [1,2,3,4,5,6]. Members of the genus are Gram-negative, aerobic, heterotrophic and dark-orange-pigmented marine bacteria. Some types of strains of the genus *Arenibacter* were recovered from various marine environments, including bottom-sediment samples, the brown alga *Chorda filum*, the green alga *Ulva fenestrata* and the edible holothurian *Apostichopus japonicus*, collected from Sea of Japan, Sea of Okhotsk and South China Sea in the Pacific Ocean.

β-*N*-Acetyglucosaminidases (EC 3.2.1.52) catalyze the hydrolytic removal of β-1,4-linked *N*-acetylglucosamine/*N*-acetylgalactosamine or only *N*-acetylglucosamine (NAG), respectively, from nonreducing ends of carbohydrates. NAG is a main structural component of chitin, a substantial constituent of bacterial peptidoglycan and lipopolysaccharides, and widespread in marine environments [7]. Chitin and its degradation derivatives are pharmaceutically valuable [8]. For example, chitoligosaccharides can stimulate the immune system to respond to microbial infections [9] and chitin monomers have been shown to act as anti-tumor agents [10], as well as to relieve the symptoms of osteoarthritis [11,12]. Furthermore, oligosaccharides containing a β-*N*-acetylglucosamine are considered to be useful building blocks for the synthesis of complex substances with important functions *in vivo*, such as cell recognition, adhesion, and communication [13].

β-*N*-Acetyglucosaminidases are widely distributed in animals and plants, in particular in terrestrial and marine microorganisms [13,14,15]. They are involved in many important physiological and pathological processes of organisms, such as cell structural integrity, energy storage, pathogen defense, viral penetration, cellular signaling, fertilization, development of carcinomas, inflammatory events and lysosomal storage diseases [16]. Certain types of bacteria utilize exogenous chitin as a nutrient producing chitinolytic enzymes. β-*N*-Acetyglucosaminidase is a component of the chitinolytic complex of microorganisms, that includes also chitinases and chitobiases and performs complete degradation and recycling of chitin, a highly abundant natural biopolymer, that is found mainly in the exoskeleton of crustaceans, insects and in the cell walls of fungi [13]. β-*N*-Acetyglucosaminidases, isolated from terrestrial microorganisms, have been applied to the preparation of a wide range of biologically and pharmaceutically significant compounds [13]. In addition, β-*N*-acetyglucosaminidases have been employed for the analysis of complex sugar chains in glycoproteins and glycopeptides, and their application as biocontrol agents, particularly against fungal pathogens of plants, has been found [17].

α-*N*-Acetylgalactosaminidase (EC 3.2.1.49) catalyzes the hydrolytic cleavage of the terminal α-*O*-glycoside-bonded residues of *N*-acetylgalactosamine from the nonreducing ends of various complex carbohydrates and glycoconjugates. Glycolipids, glycopeptides, and glycoproteins containing the structures with the *O*-glycoside core, oligosaccharides, and blood group A erythrocyte antigens are its physiological substrates [18,19,20]. Nevertheless, this amino sugar was identified among constituents of lipopolysaccharides of the cellular walls and capsules of bacteria [21]. In particular, carbohydrate moiety of lipopolysaccharide of the marine bacterium *Arenibacter palladensis* type strain KMM 3961^T^ have been shown to contain 2-acetamido-2-deoxy-d-galactose residue in tetrasaccharide repeating units: →2)-α-d-Man*p*-(1→6)-α-d-Man*p*-(1→4)-α-l-Gal*p*NAcA-(1→3)-β-d-Gal*p*NAc-(1→ [22].

α-*N*-Acetylgalactosaminidases are quite rare in marine bacteria in contrast with β-*N*-acetyglucosaminidases [14,23,24].

Arenibacters attracted our attention due to their ability to produce unique glycosidases such as α-*N*-acetylgalactosaminidase isolated from *A.* *latericius* KMM 426^T^ [25]. It has rare substrate specificity and is able to inactivate serological activity of human A red blood cells, efficiently removing terminal immunodominant α-1,3-linked *N*-acetylgalactosamine from group A structure antigen at neutral pH. The enzyme is continued to be of great interest in the medical, structural and biotechnology investigations.

We have previously shown that the some strains of the genus *Arenibacter* cannot degrade several high molecular weight natural biopolymers but they synthesized a wide range of glycosidases: α- and β-galactosidases, β-glucosidases, α-*N*-acetylgalactosaminidases, β-*N*-acetylglucosaminidases, α-fucosidases, α-mannosidases and β-xylosidases [24,25].

The aim of this paper is characteristic of glycoside hydrolases profiles of phylogenetically diverse marine bacteria of the genus *Arenibacter* isolated from different marine environments and selection of the most prospective strains for biotechnological application.

## 2. Results and Discussion

### 2.1. Phenotypic and Phylogenetic Characterization of *Arenibacter* Isolates

We isolated 16 bacterial strains belonging to the genus *Arenibacter* from diverse microbial communities associated with the various marine habitats including seaweeds, invertebrates and bottom sediments, which were collected from different locations of the Pacific Ocean (Table 1).

**Table 1:** The list of different types of *Arenibacter* strains used in this research.

Strain number	Sources and allocation places of Pacific Ocean
*Arenibacter certesii*	
KMM 3941^Т^	Green alga *Ulva fenestrata*, Troitsa Bay, Gulf of Peter the Great, Sea of Japan, Russia.
*Arenibacter* *echinorum*	
KMM 6032	Sea urchin *Strongylocentrotus intermedius*, Troitsa Bay, Gulf of Peter the Great, Sea of Japan, Russia.
KMM 6047	
*Arenibacter latericius*	
KMM 426^T^	Sediments, depth of 20 m, Ku-Lao-Re Island, South China Sea, Vietnam.
KMM 3522	Holothurian * Apostichopus japonicus*, Troitsa Bay, Gulf of Peter the Great, Sea of Japan, Russia.
KMM 3557	Holothurian * Apostichopus japonicus*, Troitsa Bay, Gulf of Peter the Great, Sea of Japan, Russia.
KMM 3523	Brown alga *Chorda filum*, Iturup Island, Sea of Okhotsk, Russia.
*Arenibacter* *palladensis*	
KMM 3961^T^	Green alga *Ulva fenestrata*, Troitsa Bay, Gulf of Peter the Great, Sea of Japan, Russia.
KMM 3980	
*Arenibacter troitsensis*	
KMM 3674^Т^	Sediments, depth of 3 m, Troitsa Bay, Gulf of Peter the Great, Sea of Japan, Russia.
KMM 6037	Green alga *Acrosiphonia sonderi*, Troitsa Bay, Gulf of Peter the Great, Sea of Japan, Russia.
KMM 6212	
KMM 6195	Brown alga *Laminaria japonica*, Troitsa Bay, Gulf of Peter the Great, Sea of Japan, Russia.
*Arenibacter* **spp.**	
KMM 6273	Sea urchin *Strongylocentrotus intermedius*, Troitsa Bay, Gulf of Peter the Great, Sea of Japan, Russia.
KMM 6684	Brown alga *Chorda filum*, Iturup Island, Sea of Okhotsk, Russia.
KMM 6685	Green alga *Ulva fenestrata*, Troitsa Bay, Gulf of Peter the Great, Sea of Japan, Russia.

All strains demonstrated similar phenotypic characteristics (Table 2). They were aerobic, heterotrophic, rod-shaped, Gram-negative, non-motile and dark-orange pigmented organisms. None of the strains showed agarase, amylase, chitinase or cellulase activities. Most strains formed acid from galactose, glucose, lactose, melibiose, raffinose and sucrose, but they could not hydrolyze casein, gelatin, DNA and Tweens.

**Table 2:** Phenotypic characteristics of *Arenibacter* strains.

Characteristic	*A. latericius*				*A. certesii*	*A. echinorum*		*A.* *palladensis*		*A. troitsensis*				*Arenibacter* spp.		
	KMM 426^T^	KMM 3522	KMM 3523	KMM 3557	KMM 3941^T^	KMM 6032^T^	KMM 6047	KMM 3961^T^	KMM 3980	KMM 3674^T^	KMM 6037	KMM 6212	KMM 6195	KMM 6273	KMM 6684	KMM 6685
Gliding motility	-	-	-	-	-	+	+	+	+	-	-	-	-	-	-	+
Na^+^ requirement	+	+	+	+	+	-	-	-	-	+	+	-	+	-	+	-
Growth with:																
8% NaCl	+	+	+	+	+	+	+	+	+	-	-	-	-	+	+	+
10% NaCl	-	-	-	-	+	-	-	+	+	-	-	-	-	-	-	+
Maximum growth temperature (°C)	42	42	42	42	38	35	32	38	38	42	37	38	42	40	42	38
Nitrate reduction	+	+	+	+	+	-	-	-	-	+	+	+	-	+	+	-
H_2_S production	-	-	-	-	-	-	-	-	-	+	-	-	-	-	-	-
Hydrolysis of:																
Casein	-	-	-	-	-	-	-	-	-	-	+	-	-	-	-	-
Gelatin	-	-	-	-	-	-	-	-	-	+	+	-	-	+	-	-
Tween 20	-	-	+	+	-	-	+	-	-	-	-	-	-	-	+	-
Tween 40	-	-	+	-	-	+	+	-	+	+	+	-	+	-	-	+
Tween 80	-	-	-	-	-	-	-	-	+	-	-	-	+	-	-	-
DNA	-	+	-	+	-	-	-	-	-	-	-	-	-	-	+	-
Urea	+	+	+	+	+	-	-	-	-	-	-	-	-	-	+	-
Acid from:																
Arabinose	-	+	+	+	-	-	-	+	-	-	-	-	-	-	+	-
Galactose	+	+	+	+	+	-	-	+	+	-	+	-	+	+	+	+
Glucose	+	+	+	+	+	+	+	+	+	-	+	+	+	+	+	+
Lactose	+	+	+	+	+	+	+	+	+	-	+	+	+	-	+	+
Melibiose	+	-	+	+	+	+	+	+	+	-	+	+	+	+	+	+
Raffinose	+	+	+	+	+	+	-	-	+	-	+	+	+	-	+	+
Rhamnose	+	-	+	-	-	+	+	+	+	-	+	+	-	+	-	+
Sucrose	+	+	+	+	+	+	+	+	+	+	+	+	+	-	+	+
Xylose	-	-	-	-	-	+	+	+	+	-	+	+	+	+	-	+
*N*-Acetylglucosamine	+	-	+	-	+	-	-	-	-	-	-	-	-	+	-	+
Glycerol	+	+	+	+	-	-	-	-	-	-	-	-	-	-	+	-
Utilization of:																
Arabinose	+	+	+	+	+	+	+	+	+	+	+	-	+	+	+	+
Mannitol	+	+	+	+	-	+	+	-	+	-	-	-	-	-	+	+
DNA G + C content (mol%)	37.5	38.0	38.2	37.9	37.7	39.4	39.2	40.2	39.2	40.0	38.0	40.2	39.9	41.7	37.7	39.4

No inhibitory activity against any of the test pathogenic cultures was observed.

**Figure 1:**
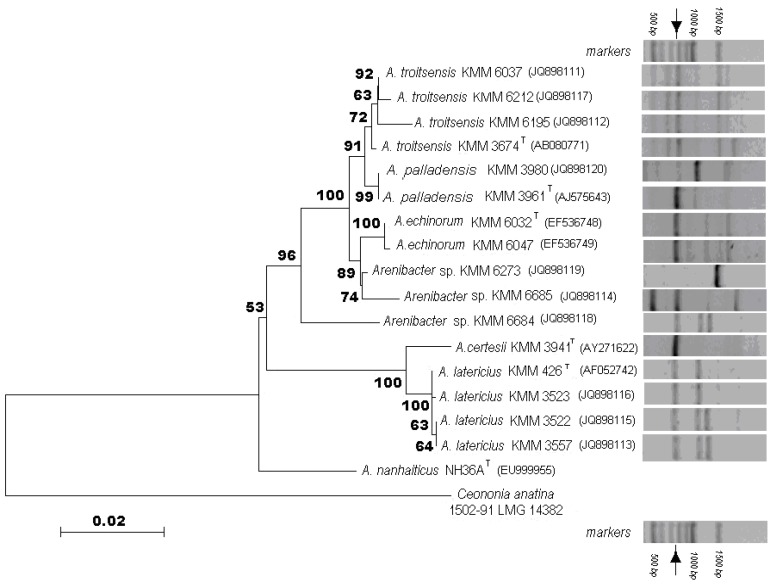
Phylogenetic Neighbor-joining tree for 16S rRNA genes showing a taxonomic position of the marine bacteria of the genus *Arenibacter* isolated from various biotopes of the South China Sea, Okhotsk Sea and Japan Sea (left). Polymerase Chain Reaction (PCR) product pattern of glycosidase-like genes of *Arenibacter* strains obtained with the use of the glycosidase-specific oligonucleotides (right). Arrow shows the position of the band corresponding to β-*N*-aсetylglucosaminidase gene.

However, phylogenetic analysis based on 16S rRNA gene sequencing revealed that the isolates divided into two main separate clusters with an evolutionary distance of approximately 5%, and those could be divided into eight different groups within the genus *Arenibacter* (Figure 1, left).

The first cluster contained two groups presented by the strains that have previously been described as members of species *Arenibacter latericius* and *A. certesii* [1,3]. Strains KMM 3522, KMM 3523, KMM 3557 and KMM 426^T^, the type strain of *A. latericius*, showed 99.9%–100% identical 16S rRNA gene sequence homology among themselves whereas these five strains were recovered from different sources collected from various areas of the Pacific Ocean (Table 1). The second cluster involved six phylogenetic groups. The strains of the third and forth groups were described as *A. echinorum* and *A. palladensis*, respectively [4,5]. A fifth phylogenetic group is presented by four strains, one of them is the type and single strain of species *A. troitsensis*, designated KMM 3674^T^ [2]. As shown in the phylogenetic tree (Figure 1, left), strains KMM 6037, KMM 6195 and KMM 6212 formed a robust cluster with strain KMM 3674^T^. While strains KMM 6037 and KMM 6212 shown a high level of the 16S rRNA gene sequence similarity of 99.6%–99.8% with *A. troitsensis* KMM 3674^T^, strain KMM 6195 had only 99.1% of sequence similarity. However, a tree topology placed strain KMM 6195 within *A. troitsensis* group (Figure 1, left). It should be noted that the novel strains significantly differed from strain KMM 3674^T^ by a set of phenotypic characteristics (Table 2). Therefore, based on these findings, the emended description of species *Arenibacter troitsensis* may be proposed. The rest of the phylogenetic groups had been of special interest because strains KMM 6273, KMM 6684 and KMM 6685 are potentially members of three distinct and novel species of the genus *Arenibacter* (Figure 1, left). Thus, strain KMM 6273 was most closely related to *A. echinorum* KMM 6032^T^, *A. palladensis* KMM 3961^T^ and *A. troitsensis* KMM 3674^T^ with 16S rRNA gene sequence similarity of 99.4%, 99.2% and 99.1%, respectively. Evolutionary distances between KMM 6273 and other known *Arenibacter* species varied from 94.6% to 96.0%. Interestingly, the novel strain and members of the species *A. echinorum* were isolated from the same sample of tissues of the sea urchin *Strongylocentrotus intermedius* (Table 1). Strain KMM 6685, recovered from the green alga *Ulva fenestrata*, also affiliated with *A. echinorum* cluster showing 16S rRNA gene sequence homology 98.8, 98.7 and 98.5% to *A. echinorum*, *A. palladensis* and *A. troitsensis*, respectively. The 16S rRNA gene sequence similarity between strain KMM 6685 and other recognized species of the genus *Arenibacter* was in range of 94.1%–96.1%. Finally, strain KMM 6684, an associate of the brown alga *Chorda filum*, occupied an intermediate position between two main phylogenetic groups, *A. troitsensis–A. echinorum* and *A. certesii–A. latericius* (Figure 1, left). The nearest recognized neighbors of strain KMM 6684 were *A. troitsensis* KMM 3674^T^, *A. palladensis* KMM 3961^T^ and *A. echinorum* KMM 6032^T^ with 97.0, 96.9 and 96.7% 16S rRNA gene sequence similarity, respectively. A level of 16S rRNA gene sequence identity between strain KMM 6684 and *A. latericius* KMM 426^T^ and *A. certesii* KMM 3941^T^, members of second main phylogenetic cluster within the genus *Arenibacter*, was 94.3%–95.0%, and with *A. nanhaiticus* NH36A^T^ it was 95.5%. Strains KMM 6273, KMM 6684 and KMM 6685 demonstrated from 96.6% to 99.2% 16S rRNA gene sequence homology to each other. This fact, in combination with the differences in phenotypic characteristics, confirmed our suggestion to place these strains in three distinct species of the genus *Arenibacter*.

### 2.2. Analysis of Glycosidase Activities of the *Arenibacter* Isolates

None of the extracts *Arenibacter* isolates had effect on polysaccharides used in this study at pH 5.2 or at pH 7.3 (Section *3.4.*—“Enzymatic Assays”). However, under physiological conditions (pH 7.3) for the bacteria growth they synthesized a wide range of glycosidases. The values of the specific activity of intracellular glycosidases of the strains measured under standard conditions are listed in Table 3.

**Table 3:** Activity of *Arenibacter* intracellular glycosidases.

Number KMM	Specific activities (mU/mg protein)								
	β- *N*-aсetyl-glucosaminidase	α- *N*-aсetyl-galactosaminidase	α-fucosidase	α-galactosidase	α-glucosidase	α-mannosidase	α-xylosidase	β-galactosidase	β-glucosidase
*A. latericius*									
426^T^	96.3 ± 7.1 *	12.0 ± 0.9	3.4 ± 0.4	0.77 ± 0.04	1.11 ± 0.07	0.49 ± 0.04	0.66 ± 0.04	0.024 ± 0.005	0.3 ± 0.03
522	122.5 ± 4.8	27.3 ± 1.3	4.04 ± 0.02	1.61 ± 0.09	4.82 ± 0.19	2.54 ± 0.18	0.68 ± 0.03	1.78 ± 0.13	1.53 ± 0.03
3557	143.1 ± 4.3	25.6 ± 3.8	8.23 ± 0.4	0.23 ± 0.03	1.65 ± 0.04	2.06 ± 0.09	0.28 ± 0.01	0	1.56 ± 0.17
3523	113.29 ± 1.54	18.53 ± 0.24	7.64 ± 0.04	0.28 ± 0.004	3.86 ± 0.14	2.17 ± 0.17	1.23 ± 0.11	2.51 ± 0.26	1.27 ± 0.04
*A. certesii*									
3941^Т^	204.81 ± 4.03	24.45 ± 1.42	1.7 ± 0.12	0	1.11 ± 0.10	1.43 ± 0.11	0	0	1.58 ± 0.13
*A. echinorum*									
6032^T^	60.13 ± 2.43	11.93 ± 0.57	8.48 ± 0.48	0.62 ± 0.06	0.91 ± 0.19	0.82 ± 0.05	0.34 ± 0.09	1.5 ± 0.13	1.58 ± 0.19
6047	125.5 ± 1.98	25.86 ± 2.32	10.38 ± 0.23	0.7 ± 0.12	2.08 ± 0.05	1.88 ± 0.048	0.51 ± 0.07	0.79 ± 0.04	1.58 ± 0.09
*A. palladensis*									
3961^T^	50.97 ± 0.89	14.06 ± 0.68	5.5 ± 0.6	0.91 ± 0.09	0.56 ± 0.05	1.21 ± 0.10	0	0	0.84 ± 0.025
3980	25.85 ± 1.60	2.26 ± 0.28	2.36 ± 0.38	1.81 ± 0.11	0.77 ± 0.14	0.48 ± 0.08	0	0	0
*A. troitsensis*									
3674^Т^	26.91 ± 1.65	7.9 ± 0.36	2.18 ± 0.19	0	0.64 ± 0.04	3.34 ± 0.28	0	0	0
6037	24.4 ± 2.3	4.86 ± 0.71	3.62 ± 0.41	0	0.93 ± 0.14	0.25 ± 0.06	0	0	0
6195	23.81 ± 0.41	7.9 ± 0.3	2.71 ± 0.27	0.52 ± 0.09	0.35 ± 0.02	0.63 ± 0.05	0.36 ± 0.05	0	1.36 ± 0.18
6212	24.05 ± 0.49	5.31 ± 0.14	3.19 ± 0.3	0.68 ± 0.06	0	1.51 ± 0.11	0	0	0
*Arenibacter* **spp.**									
6273	20.53 ± 1.08	5.73 ± 0.21	3.04 ± 0.34	2.71 ± 0.26	3.10 ± 0.38	2.93 ± 0.23	0.90 ± 0.04	2.43 ± 0.22	2.0 ± 0.12
6684	96.77 ± 1.58	36.42 ± 0.88	4.73 ± 0.40	3.93 ± 0.32	5.7 ± 0.1	5.3 ± 0.62	1.49 ± 0.04	3.13 ± 0.67	6.01 ± 0.96
6685	13.71 ± 1.24	2.24 ± 0.22	2.29 ± 0.21	4.11 ± 0.87	1.60 ± 0.12	0.18 ± 0.01	0.28 ± 0.06	0.10 ± 0.03	0.09 ± 0.02

* Glycosidase activity in the extracts of bacteria was calculated as the average of three independent experiments.

All isolates synthesized β-*N*-aсetylglucosaminidases, α-*N*-aсetylgalactosaminidases, α-fucosidases and α-mannosidases, but the enzymes demonstrated different levels of a specific activity. It should be noted that none of the representatives of *A. certesii*, *A.* *palladensis* and *A. troitsensis* possessed significant α-xylosidase and β-galactosidase activities under conditions studied.

Highly active β-*N*-acetylglucosaminidases were found to be the main glycosidase for all *Arenibacter* strains irrespective of the isolation sources and geographic locations (Table 3). The differences were observed only among the level of the enzyme expression. Highest activity of β-*N*-acetyglucosaminidase was observed in *Arenibacter certesii* KMM 3941^T^, isolated from a green alga *Ulva fenestrata*. An equally high level of β-*N*-acetylglucosaminidase activity was found in *A. latericius* KMM 3522 and KMM 3557, isolated from a sea cucumber *Apostichopus japonicus*, as well as *A. latericius* KMM 3523, isolated from brown alga *Chorda filum*. In *A. echinorum* KMM 6047 from the sea urchin *Strongylocentrotus intermedius* and *Arenibacter* sp. KMM 6684 from brown algae *Chorda filum* the level of β-*N*-acetylglucosaminidase activity was slightly lower (Table 3). At the same time, all strains of phylogenetically closely related species *A. troitsensis* and *A. palladensis* were characterized by a lower level of activity of this enzyme than the above strains (Table 3).

Although little is known about chitin degradation in marine bacteria, it has been previously reported that *Gammaproteobacteria* of the genus *Alteromonas* and *Vibrio* possessed all kinds of chitinolytic enzymes [13,26,27,28,29]. Surprisingly, no chitinases cleaving polymer to chitooligosacchrides as well as other polysaccharases were observed in any *Arenibacter* strains explored belonging to the phylum *Bacteroidetes*. However, the presence of β-*N*-acetyglucosaminidases, other key enzymes of chitinolytic complex in all strains, allow the suggestion that arenibacters can participate in the second step of chitin degradation, cleaving *N*-acetylglucosamine from non-reducing termini of chitooligosaccharides obtained after the action of endo-chitinases for other members of the microbial community on the insoluble chitin. Moreover, five strains, including KMM 426^T^, KMM 3523, KMM 3941^T^, KMM 6273 and KMM 6685, demonstrated the ability to oxidize *N*-acetylglucosamine, which can serve as a source of carbon and nitrogen (Table 2).

α-*N*-Aсetylgalactosaminidase was glycosidase of the second level of activity in all *Arenibacter* strains. The ratio of β-*N*-aсetylglucosaminidases/α-*N*-aсetylgalactosaminidases activities were not above 10:1 for all strains studied (Table 3).

However, marine bacteria of the phylum *Bacteroidetes*, especially of genus *Arenibacter*, are the best producers of the enzyme [24]. *A.* *latericius* KMM 426^T^ have previously been shown to synthesize some molecular forms of α-*N*-atcetylgalactosaminidase. Previously, we isolated and characterized one of them as being biotechnologically important, as it removed serological activity of human A red blood cells [25].

#### Structural Characteristics of *A.* *latericius* α-*N*-acetylgalactosaminidase

*N*-terminal sequence of the enzyme purified to homogeneity using the procedure previously described [25] was GAKYMGGFSAPKLDT. The molecular masses of the enzyme were 48 ± 2 and 94 ± 3 kDa, as determined by SDS-PAGE and analytical size, exclusion, fast performance, liquid chromatography, respectively. These results revealed that the enzyme is a homodimer protein in solution.

### 2.3. Molecular Genetic Analysis of Glycosidases

In this study, we employed a homology-based strategy to isolate genes of glycoside hydrolases from genomes of *Arenibacter* isolates. Several sets of oligonucleotide primers were designed based on the fully-characterized and putative genes of GH20 (Clan GH-K), GH27, GH36 (Clan GH-D) and GH109 enzymes from bacteria, fungi and eukaryotes recovered from the GenBank database. Evolutionary relationships were established between GH20, GH27 and GH36 proteins. Enzymes of the clans GH-D and GH-K share the most important functional characteristics, such as composition of the active center, anomeric configuration of cleaved glycosidic bonds, and retaining mechanism of the catalyzed reaction. Proteins of these clans have the same three-dimensional structure of catalytic domains as (β/α)_8_ and common evolutionary origin of their genes [30]. However, only two PCR products, namely GH20 β-*N*-acetylglucosaminidase and GH109 α-*N*-acetylgalactosaminidase, were successfully amplified from the genomic DNA of *Arenibacter* strains. No genes of GH27 and GH36 proteins were found.

The PCR-products pattern with the set of GH20-specific oligonucleotides was found to reflect the different types of *Arenibacter* strains, corresponding to the 16S rRNA gene analysis results (Figure 1, right). The PCR resulted in three to six bands per sample. The major bands for all *Arenibacter* isolates with the length of about 750–800 bp were corresponded to the partial sequence of β-*N*-acetyglucosaminidase genes (Figure 1, right). The other bands bearing unidentified proteins suggest that there may be a large undiscovered metabolic capacity of *Arenibacter*.

#### 2.3.1. Band Pattern Analysis

According to the band pattern analysis, *Arenibacter* isolates were divided into 10 groups:

- *A. latericius* KMM 426^T^ and KMM 3523;
- *A. latericius* KMM 3522 and KMM 3557, *Arenibacter* sp. KMM 6684;
- *A. troitsensis* KMM 6037, KMM 6212 and KMM 6195;
- *A. troitsensis* KMM 3674^T^;
- *A. palladensis* KMM 3980;
- *A. palladensis* KMM 3961^T^;
- *A. echinorum* KMM 6032^T^ and KMM 6047;
- *A. certesii* KMM 3941^Т^;
- *A. Arenibacter* sp. KMM 6273;
- *A. Arenibacter* sp. KMM 6685.

The results of the detailed taxonomic study showed that strains of the genus *Arenibacter* were phylogenetic and metabolic diverse organisms that were found in various marine microbial communities associated with seaweeds, invertebrates and sediments. Most of the isolates were affiliated with known species *Arenibacter* *latericius*, *A. certesii*, *A. echinorum*, *A.* *palladensis* and *A. troitsensis*. Three newly sequenced strains occupied distinct phylogenetic positions among the recognized *Arenibacter* species (Figure 1). Most of the strains explored belonged to the two separated phylogenetic clusters that formed by members of species *A. troitsensis*–*A. paladensis*–*A. echinorum* and *A. certesii*–*A. latericius*, respectively*.* Interestingly, despite significant phylogenetic distances, a novel strain KMM 6684, isolated from the Okhotsk Sea brown alga, and members of *A. latericius*, associated with holothurians from Sea of Japan, displayed similar glycosidase spectra and band patterns (Table 3, Figure 1). At the same time, four *A. latericius* strains having the comparable profiles of glycoside hydrolases (Table 3) were divided in to two groups according to the results of the band patterns. The first group incorporated isolates of holothurians KMM 3522 and KMM 3557, the next group included two other strains, KMM 426^T^ and KMM 3523, isolated from bottom sediment of South China Sea and brown alga of Okhotsk Sea, respectively (Figure 1). *A. troitsensis* and *A. palladensis* isolates were grouped together, which was in agreement with their taxonomic positions and glycosidase activity profiles (Figure 1; Table 3). However, *A. troitsensis* and *A. palladensis,* as well as *A. latericius,* band patterns were divided into two groups. Thus, glycosidase-encoding genes analysis using phylogenetic information was suggested to be suitable for providing a characterization of members of the genus *Arenibacter* in the natural microbial populations, their diversity prediction in the environmental samples and elucidation of their possible ecological role in the marine environment.

#### 2.3.2. GH20 β-*N*-acetylglucosaminidase of *Arenibacter* Isolates

Sequence analysis of the major 750 bp-length bands of *Arenibacter* isolates suggested that the resultant DNA fragments were new GH20 members (Figure 1, right). All *Arenibacter* strains demonstrated 99.9%–100% β-*N*-acetyglucosaminidase gene sequence homology to each other (data not shown).

**Figure 2:**
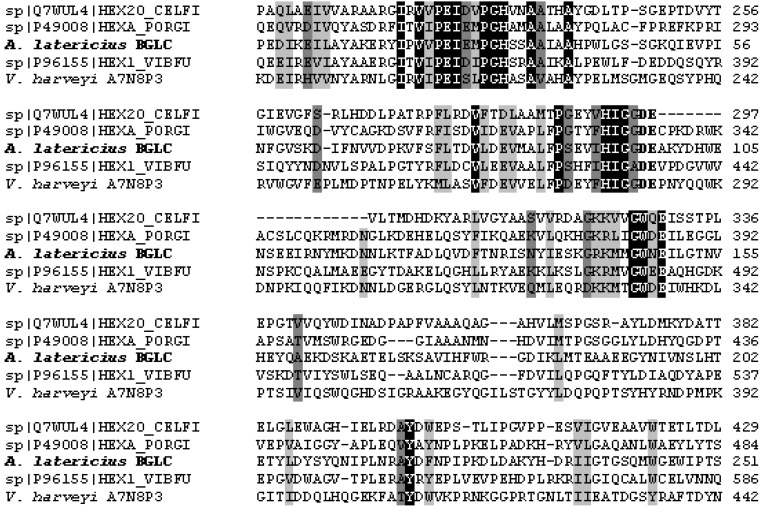
Alignment of a partial *A. latericius* β-*N*-acetyglucosaminidase (BGLC) amino acid sequence with other characterized GH20 family β-hexosaminidases: *Vibrio furnissii* ExoI, HEX1_VIBFU (P96155), *Vibrio harveyi* chitobiase (A7N8P3), *Porphyromonas* g*ingivalis* β**-**hexosaminidase, HEXA_PORGI (P49008), and *Cellulomonas fimi* β**-**hexosaminidase, HEX20_CELFI (AF478459). Identical residues are indicated by black and similar residues by darkly grey and lightly grey. The acidic pair important for enzyme catalysis is in bold font.

BLAST search indicated high sequence similarity of *Arenibacter* β-*N*-acetyglucosaminidases genes to the ones from other marine *Bacteroidetes* species, among them *Polaribacter* sp. MED 152 was dominant (80% homology). It should be noted that most of these sequences turned out to be only hypothetical proteins with predicted activity. A search of the Swiss-Protein Data Bank identified several characterized β-*N*-acetyglucosaminidases with significant similarities to the translated open reading frames of the β-*N*-acetyglucosaminidase gene of *Arenibacter* (Figure 2).

These proteins include: *Porphyromonas gingivalis* β-*N*-acetylhexosaminidase, β-Nahase (34% identity and 52% homology); *Vibrio furnissii* β-*N*-acetyglucosaminidase, ExoI (31% identity and 48% homology); *Cellulomonas fimi* β-*N*-acetylhexosaminidase, Hex20 (28% identity and 41% homology); the lysosomal β-chain and α-chain of human β-hexosaminidase (24% dentity and 46% homology) [27,31,32]. The same similarity was observed with *Vibrio harveyi* chitobiase, encoded by VIBHAR_06345 gene [29].

As seen in Figure 2, several consensus residues identified may participate in the catalytic function of the *Arenibacter* β-*N*-acetyglucosaminidases. In accordance with sequence-based CAZy classification all well-characterized marine bacterial β-*N*-acetyglucosaminidases are related to the GH20 family of Clan GH-K and catalyzed the hydrolysis of *O*-glycoside bond with retention of anomeric configuration [33,34]. GH20 enzymes employ a “substrate-assisted” mechanism involving the transient formation of an oxazolinium ion intermediately required for action of the catalytic nucleophile/base-carbonyl oxygen of C-2 acetamido group of substrate and catalytic proton donor–Glu [35]. The GH20 enzymes show significant sequence homology to each other but differ in its substrate specificity. For example, Tsujibo *et al.* purified and characterized a transglycosylating GH20 enzyme from *Alteromonas* sp. strain O-7 which synthesized β-(1→6)-(GlcNAc)_2_, 2-acetamido-6-*O*-(2-acetamido-2-deoxy-β-d-glucopyranosyl)-2-deoxyglucopyranose from β-(1→4)-(GlcNAc)_2_ [26]. At the same time, the active site of *V*. *furnissii* is of the second type of β-*N*-acetyglucosaminidase that contains three to five GlcNAc binding subsites, depending on the substrate specificity of individual enzymes [27]. ExoI of *V. furnisii* can act as a chitobiase, but only at non-physiological pH values (pH 5.8). It remains to be seen whether this large homology of the *Arenibacter* β-*N*-acetyglucosaminidase and the aligned *V. furnissi* ExoI, *V. harveyi* chitobiase, *P.* *g*ingivalis and *C. fimi* enzymes will extend to the whole protein.

#### 2.3.3. GH109 α-*N*-acetylgalactosaminidase from *Arenibacter latericius*

The similar identity and homology was observed in the alignment of full-length *Arenibacter latericius* α-*N*-acetylgalactosaminidase (acession no.HQ108058) with the GH109 proteins from the same bacteria mainly deduced from the metagenomic projects. The closely related proteins were from the genomes (a Swiss-Prot search): *Akkermansia muciniphila* (66% identity, 80% homology); *Shewanella oneidensis* (55% identity, 70% homology); *Streptomyces erythraeus* (*Saccharopolyspora erythraea*) (39% identity, 58% homology); *Bacteroides vulgatus* (36% identity, 56% homology); *Flavobacterium meningosepticum* (37% identity, 56% homology); *P. gingivalis* (35% identity, 55% homology). It is evident, that the most homologous are found among the predicted proteins. There are five GH109 α-*N*-acetylgalactosaminidase that are characterized in terms of properties and substrate specificity [36]. However, α-*N*-acetylgalactosaminidase of the human pathogen *F. meningosepticum* (*Elizabethkingia meningoseptica*) belonging to the phylum *Bacteroidetes* is still a comprehensively characterized GH109 enzyme with established crystal structure [36].

**Figure 3:**
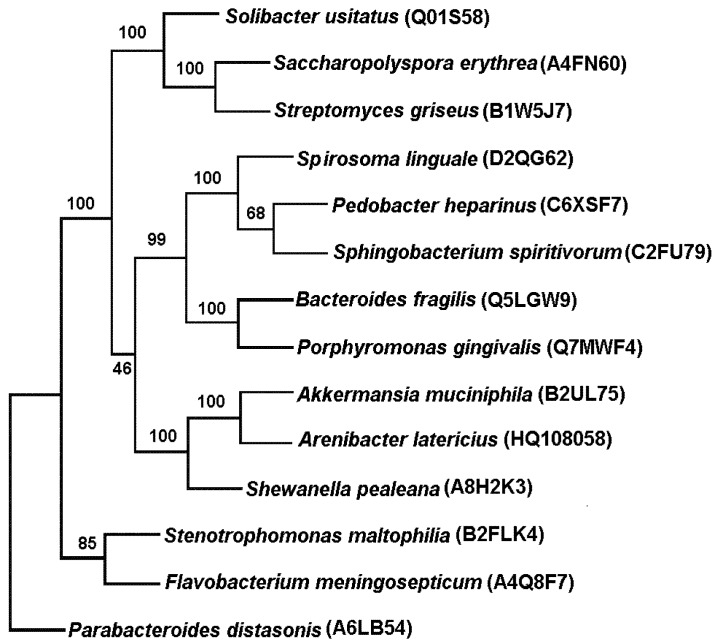
Consensus tree of *A. latericius* α-*N*-acetylgalactosaminidase GH109-like amino acid sequences was reconstructed using the protein maximum likelihood method implemented in the PHYLIP program (v3.6) [37]. Reliability for internal branch was assessed using the bootstrapping method (100 bootstrap replicates). Graphical representation of the phylogenetic tree was performed with Drawtree from the PHYLIP package (v3.6) [37]. Amino acid sequences of α-*N*-acetylgalactosaminidases were aligned using MUSCLE v3.7 [38].

Phylogenetic analysis showed that α-*N*-acetylgalactosaminidase of marine bacterium *Arenibacter latericius* KMM 426^T^ belongs to GH109 enzymes and was most closely related to the predicted protein structures of *Akkermansia* *muciniphila*, an inhabitant of the human intestinal tract, affiliated with the phylum Verrucomicrobia, and marine gammaproteobacterium *Shewanella pealeana* (Figure 3).

GH109 enzyme, isolated from a clinical strain *Elizabethkingia meningoseptica* (formerly [*Flavobacterium*] *meningosepticum*), which is also the representative of the phylum *Bacteroidetes*, evolutionarily distant from the α-*N*-acetylgalactosaminidase of *Arenibacter* *latericius*, though descended from the same ancestor. Both enzymes have a common ancestor with the oxidoreductases that perform very different functions and have high homology with GH109 enzymes. GH109 α-*N*-acetylgalactosaminidase catalyzes the hydrolysis of *O*-glycoside bond with retention of anomeric configuration and reveals a catalytic mechanism involving NAD^+^, unusual for the classic glycoside hydrolases, proposed by Koshland [39]. GH109 α-*N*-acetylgalactosaminidase of *Elizabethkingia meningoseptica* is capable to efficient removal of A antigens at neutral pH with low consumption of recombinant enzymes.

According to BLAST search results, the highest gene sequence similarity of *A. latericius* α-*N*-acetylgalactosaminidase was found with the predicted proteins from the other marine *Bacteroidetes*: *Zobellia galactanivorans*, *Maribacter* sp. and *Muricauda ruestringensis* (69%–83% identity, 82%–92% homology) (data not shown). It is an interesting fact that this list was followed by the human symbiotic gut colonizer, *Akkermansia muciniphila*, breaking down extracellular polymeric substrates, including mucin [40]. However, *A. latericius* is an aerobic marine bacterium found in a wide variety of environments including sediments, animals and alga. This suggests that *Arenibacter* can be a marine host associate, as well as attach to solid surfaces and form biofilms. The wide spectrum of glycoside hydrolyses can play a role in facilitating detachment and dispersion of *Arenibacter* cells for successful colonization of new surfaces. It is within the realm of possibility that *Arenibacter* lifestyle includes participation in degradation of the remains of other organisms such as chitin or plant biopolymers, making them important organisms in carbon recycling. Indeed, it has been previously reported that marine planktonic *Flavobacteria* (dominated by *Polaribacter*) have been defined to attach to, and to degrade, diverse complex algal organic material and then make labile compounds available to *Alphaproteobacteria* and *Gammaproteobacteria* [41]. It is evident that many of the *Arenibacter* multienzyme complex features are niche and are habitat adaptations that facilitate depolymerization of complex polysaccharides in the marine environment. *Arenibacter* can be responsible for producing natural unique glycosides useful for biotechnology and human medicine.

## 3. Materials and Methods

### 3.1. Strains Isolation and Purification

The strains were isolated from bottom sediments; the green algae *Acrosiphonia sonderi* and *Ulva fenestrata*, the brown algae *Chorda filum*, the sea urchin *Strongylocentrotus intermedius* and the holothurian *Apostichopus japonicus* collected in Troitsa Bay, Gulf of Peter the Great, the Sea of Japan (Table 1). For strains isolation, 0.1 mL bottom sediment suspension or tissue homogenates were transferred onto marine agar 2216 (Difco) plates. After primary isolation and purification, strains were cultivated at 28 °C on the same medium and stored at −80 °C in marine broth (Difco) supplemented with 20% (v/v) glycerol.

### 3.2. Morphological, Biochemical, and Physiological Characterization

The physiological, morphological and biochemical properties of the strains were studied using the standard methods. Gram-staining was performed as recommended by Smibert *et al.* [42]. Oxidative or fermentative utilization of glucose was determined on the Hugh-Leifson medium modified for marine bacteria [43]. Catalase activity was tested by addition of 3 % (v/v) H_2_O_2_ solution to a bacterial colony and observation for the appearance of gas. Oxidase activity was determined by using tetramethyl-*p*-phenylenediamine. Degradation of agar, starch, casein, gelatin, chitin, Tweens 20, 40 and 80, DNA and urea, growth at different pH values and production of acid from carbohydrates, nitrate reduction, production of hydrogen sulphide, acetoin (Voges–Proskauer reaction) and indole, and presence of alkaline phosphatase activity were tested according to standard methods [42]. The temperature range for growth was assessed on MA. Tolerance to NaCl was assessed in medium containing 5 g Bacto Peptone (Difco), 2 g Bacto Yeast Extract (Difco), 1 g glucose, 0.02 g KH_2_PO_4_ and 0.05 g MgSO_4_·7H_2_O per liter of distilled water with 0, 0.5, 1.0, 1.5, 2.0, 2.5, 3, 4, 5, 6, 8, 10 and 12% (w/v) of NaCl. Carbon source utilization was tested (i) using commercial API 20E (bioMérieux) identification strip following the instructions of the manufacturer, and (ii) using a medium that contained 0.2 g NaNO_3_, 0.2 g NH_4_Cl, 0.05 g Yeast Extract (Difco) and 0.4% (w/v) carbon source per liter of artificial seawater as described by Suzuki *et al.* [44].

For mol% G + C determination, DNA was isolated following the method of Marmur [45] and the DNA G + C content was determined by the thermal denaturation method [46].

#### Antimicrobial Activity

To order to determine antimicrobial activity of the isolates, the following test organisms were used: *Bacillus subtilis* ATCC 6633^T^, *Enterococcus faecium* LMG 11423^T^ and *Staphylococcus aureus* ATCC 21027^T^ as the Gram-positive strains, *Escherichia coli* 3254, *Pseudomonas aeruginosa* ATCC 27853^T^ and *Vibrio parahaemolyticus* CIP 75.2^T^ as the Gram-negative strains, and *Candida albicans* KMM 455 as the yeast strain. All strains were grown on tryptic soy agar (TSA) plates with adding of NaCl (15 g/L) at 37 °С. The agar plates were inoculated by using overnight cultures of each test strain with approximately 10^9^ cells per milliliter (100 μL for the bacterial strains and 200 μL for the yeast strain). The *Arenibacter* isolates were grown as a lawn on MA at 28 °C for 24 h. Plugs 10 mm in diameter were cut out with a chokbore and placed with the bacterial side down onto test strains agar plates. After incubation at 30 °С for 24 h, antimicrobial activity was evaluated by measuring the inhibition zones (in mm) around the agar plugs.

### 3.3. Protein Assays

Protein concentration in bacterial biomass extracts was determined according to the Bradford [47] using bovine serum albumin (Sigma) as the standard. α-*N*-acetylgalactosaminidases was isolated, purified and identified according procedure described earlier [25]. The NH_2_-terminal amino acid sequence (15 a.a.) of the α-*N*-acetylgalactosaminidases was determined using a pulsed liquidphase protein sequencer (Procise 492; Applied Biosystems Foster City, CA, USA). Sodium dodecyl sulfate polyacrylamide gel electrophoresis (SDS-PAGE) was run essentially as described by the supplier of the electrophoresis equipment (Hoefer Scientific Instruments, San Francisco, CA, USA). The molecular mass of native α-*N*-acetylgalactosaminidases was estimated by gel filtration on a Superose 12 HR 10/30 column (Amersham Pharmacia) running fast protein liquid chromatograph (Acta, France) in 0.05 M Na^+^-phosphate, рН 7.3, 0.15 M NaCl at a flow of 0.4 mL/min at 8 °С and calibrated using gel filtration standard molecular weight markers (Sigma). The subunit molecular mass of the purified α-*N*-acetylgalactosaminidases was determined by 14% SDS-PAGE molecular-weight markers (Fermentas).

### 3.4. Enzymatic Assays

For enzyme activities evaluation, bacteria were cultivated on shaker at 150 rev/min for 24 h at 25 °C in 250 cm^3^ flasks, containing 50 mL of the following composition (g/L): bactopepton—5.0, yeast extract—2.0, glucose—1.0, KH_2_PO_4_—0.02 and MgSO_4_·7H_2_O—0.05 in 50% natural sea water.

For bacterial extract preparation, the cells were separated from the culture medium by centrifugation at 3000 *g*. The bacterial biomass was frozen at −20 °C. The weighed portion of the frozen raw biomass was resuspended in 0.01 M Na^+^ phosphate buffer solution, pH 7.3, on an ice bath up to concentration of 0.2 g/mL. The cells were homogenized by sonification at a frequency of 22 kHz and a current of 0.4 A three times for 20 s at intervals of 20 s. The cell suspension was incubated at 4 °C for 3 h; the homogenate was then centrifuged at 11,000 *g* for 30 min. The pellet was discarded; the protein concentration and glycosidase activities were determined in the extracts.

Glycosidase activity in bacterial extracts was measured as follows: 0.05 mL of the extract solution and 0.35 mL of *p*-nitrophenyl-glycoside solution (1 mg/mL) in 0.1 M Na^+^ phosphate buffer, pH 7.3, was incubated for 5 to 120 min at 20 °C. The reaction was stopped by addition of 0.6 mL of 1 M Na_2_CO_3_. The initial reaction rate was determined from the linear segment of the A_400_ time dependence. The substrate solution and extract solution in the same buffer with 1 M of Na_2_CO_3_ served as the controls. The amount of released *p*-nitrophenol was determined spectrophotometrically by measuring OD at 400 nm (ε_400_ = 18,300 mol^–1^·cm^–1^). One unit of activity was defined as the amount of an enzyme that releases 1 μmol of *p*-nitrophenol per minute under the conditions used. Specific enzyme activity was estimated as units (U) per milligram protein.

The polysaccharide hydrolase and lyase activities were measured in 0.05 M Na^+^-phosphate buffer, pH 7.3, at 20 °C. The reaction was carried out alternatively in 0.05 M Na^+^-acetate buffer, рН 5.2, at 20 °C for 24 h. The reaction mixture contained 0.05 mL of 0.2 g/mL bacterial cell extracts and 0.2 mL of 0.1% corresponding substrate solution in 0.05 M Na^+^-phosphate or Na^+^-acetate buffer and 0.1 M NaCl, pH 7.3 and 5.2, respectively. The reaction was terminated by adding Nelson reagent. One unit of activity was defined as the quantity of reducing sugars released from substrate using corresponding monosaccharide as a standard Somogyi–Nelson method [48].

*p*-Nitrophenyl-α- and β-d-galactopyranosides, *p*-nitrophenyl-α- and β-d-glucopyranosides, *p*-nitrophenyl-α-*N*-acetylgalactosaminide, *p*-nitrophenyl-α-l-fucopyranoside, *p*-nitrophenyl-α-d-mannopyranoside, *p*-nitrophenyl-α-xyloside (Sigma, USA) and *p*-nitrophenyl-β-*N*-acetylglucosaminide (Chemapol, Czech Republic) in concentration 1.0 mg/mL were used as substrates for α- and β-galactosidases, α- and β-glucosidases, α-*N*-acetylgalactosaminidase, α-fucosidase, α-mannosidase, α-xylosidase and β-*N*-acetylglucosaminidase, respectivaly.

Dextran, water-soluble agar, amylose, carboxymethyl cellulose (Sigma, USA), polyguluronic acid (British Drug Houses, UK), fucoidans from *Fucus evanescence* (α-1,3;1,4-l-fucan sulfate) and *Laminaria cichorioides* (α-1,3-l-fucan sulfate) free of polyphenol or alginic acid [49], pustulan from lichen *Umbilicaria rossica* (β-1,6-d-glucan [50]), laminaran from *Laminaria cichorioides* (branched β-1,3;1,6-d-glucan), polymannuronic acid from *Alaria fistulosa* (β-1,4-glycoside-bound mannuronic acid [51]), and pullulan from *Aureobasidium pullulans* (α-1,4;1,6-glucan) were used as substrates for polysaccharide degrading enzymes.

### 3.5. DNA Preparation and PCR Amplification

Genomic DNA was extracted from cultured marine bacteria using of commercial kit according to manufacturer’s instruction (Fermentas). Fresh bacterial culture (200 μL) was used for DNA isolation and 20–50 μL of water was added for final DNA dissolving.

For PCR amplification, Encyclo Polymerase and Taq Polymerase (Evrogen) were used. Two universal oligonucleotide primers were used for marine bacteria 16S rRNA gene sequence analyses (forward 11F: 5′-AGAGTTTGATC(C/A)TGGCTCAG-3′ and reverse 1492R: 5′-TACGG(C/T)TACCTTGTTACGACTT-3′).

The oligonucleotide primers used for amplification of *Arenibacter* families GH 20, GH 27 and GH 36 genes were for the forward primers:

(1) 5′-CC(T/G)CA(A/G)ATGGG(N)TGGAA-3′,
(2) 5′-ACTCC(G/A/T)ATGGGNTG-3′,
(3) 5′-CC(G/C/T)ATGGGNTT(T/C)AA(T/C)AA(T/C)TGG-3′,
(4) 5′-(A/G)TNGA(T/C)GA(T/C)GGNTGGTT-3′;

and for the reverse primers:

(5) 5′-AA(G/A)TCNGG(G/A)TC(G/A)TT(G/A)AA-3′,
(6) 5′-CAT(G/A)TCNCC(G/A)TC(A/G)TTCCA-3′,
(7) 5′-C(T/G)(G/A)TT(G/A/C)A(T/A)(G/A)TCCCA(C/T)TT-3′,
(8) 5′-AT(A/C/T)CCCAT(G/A)TGNCC(G/A)AA-3′.

Sets of oligonucleotides designed based on alignments of families GH20, GH27 and GH36 ORFs of closely related bacteria, fungi and eukaryotes in the GenBank database were: (1) and (5), (2) and (5), (3) and (5), (4) and (7), (4) and (8). PCR amplification was performed in a total volume of 20 μL mixture, containing 50 ng of chromosomal DNA, 2 μm primers, 1 mm each deoxyribonucleoside triphosphate, 5 U of Encyclo Polymerase. Samples were amplified for 35 cycles using the following program: initial denaturation at 95 °C for 2 min, denaturation at 95 °C for 15 s, annealing temperature (varying from 50 °C to 56 °C) for 15 s and elongation at 72 °C for 1.5 min.

The partial sequence of the genes encoded GH20 β-*N*-acetylglucosaminidase were amplified by the set of oligonucleotides (1) and (5) with the annealing temperature 56 °C.

The oligonucleotide primers designed on the base of the ORFs of GH109 α-*N*-acetylgalacto saminidases in the GenBank were:

Nend-acetyl_forward1—5′-GG(G/A/T)GC(A/T)AA(A/G)TA(T/C)ATGGGNGGNTT(T/C)TC-3′,
Nend-acetyl_forward2—5′-AA(A/G)TA(T/C)ATGGGNGGNTT(T/C)-3′,
*N*-acetyl_forward1—5′-CA(T/C)GCNTT(T/C)GTNGA(A/G)GTNCC-3′,
*N*-acetyl_forward2—5′-ATGATGATGGA(A/G)AA(T/C)GTNAA(T/C)TA-3′,
*N*-acetyl_reverse1—5′-CCNCTNGT(G/A)AA(G/A)TCNGG(G/A)AA-3′,
*N*-acetyl_reverse2—5′-GG(A/G)TG(G/A)TC(G/A)TA(C/T)TTNTC-3′.

The partial sequence of GH109 α-*N*-acetylgalactosaminidase was determined by oligonucleotides *N*-acetyl_forward2, 5′-ATGATGATGGA(A/G)AA(T/C)GTNAA(T/C)TA-3′, for the forward primer and *N*-acetyl_reverse1, 5′-CCNCTNGT(G/A)AA(G/A)TCNGG(G/A)AA-3′, for the reverse primer. The *C*-terminal amino acid sequence of α-*N*-acetylgalactosaminidase was amplified using forward oligonucleotide primer compatible with the partial sequence of GH109 α-*N*-acetylgalactosaminidase—5′-GTAGCACAAAATGGAGC-3′.

The fully-length genes encoded GH109 α-*N*-acetylgalactosaminidases were amplified by the following oligonucleotides within the inserts in pET40 and TEV protease site for removal His-tags: 5′-TAACCATGGGTGGGGCTAAGTACATGGGCGGTTTTTCTGCT-3′ for the forward primer and 5′-TAAGTCGACACCCTGAAAATAAAGATTTTCGCTTACAATATCTAATGGTGCAGTGGT-3′ for the reverse primer. The forward oligonucleotide primer was designed on the base of the *N*-terminal amino acid sequence of *Arenibacter latericius* α-*N*-acetylgalactosaminidase. 

PCR-products were purified with purification kit (Qiagen, USA) then cloned and sequenced.

Amplified fragments were either cloned or directly sequenced using the automated PE/ABI 310 DNA sequencer and the PE/ABI-ABI PRISM BigDye Terminator cycle sequencing Ready Reaction Kit (PE Applied Biosystems). PCR products were identified using 1% agarose gel and visualization computer system (Herolab, Germany).

### 3.6. PCR-Product and Sequence Analysis

The nucleotide sequences were edited using the software Chromas, Gene Runner and compared to published sequences in the NCBI GenBank using the nucleotide and protein BLAST and ClustalW2. Neighbor-joining (NJ) trees for 16S rRNA genes were generated from the corresponding matrix of nucleotide divergence between sequences using the program MEGA2 [52]. Confidence in the branching points was obtained with 1000 bootstrap replications. The sequence of *Coenonia anatina* LMG 1502-91 was used as outgroup for phylogenetic reconstruction. Pairwise sequence similarities were calculated by Ez-Taxon-e [53].

The phylogenetic tree was reconstructed using the protein maximum likelihood method implemented in the PHYLIP program (v3.6). Reliability for internal branch was assessed using the bootstrapping method (1000 bootstrap replicates). Graphical representation of the phylogenetic tree was performed with Drawtree from the PHYLIP package (v3.6) [37].

The resultant PCR-products obtained with the use of glycosidase-specific primers were analyzed by standard gel electrophoresis as band patterns. To reduce possible inter-sample PCR variation, all PCR reactions were run in triplicates and pooled together before loading on gel-electrophoresis and PCR amplification, and genomic DNA of *E. coli* was used as positive control, and PCR mixture without DNA template was used as negative control.

## 4. Conclusions

As a result of multifaceted research, we can draw the following conclusions. Marine bacteria of the genus *Arenibacter* are emerging as a recently discovered group of marine bacteria. The hydrolytic enzyme profiles of *Arenibacter* isolates include biotechnologically important β-*N*-aсetylheglucosaminidases, α-*N*-aсetylgalactosaminidases, α-fucosidases and α-mannosidases with the different levels of enzymatic activity. Molecular genetic analyses with use of the glycosidase-specific primers show the absence of classic GH27 and GH36 genes except for GH20 β-*N*-aсetylglucosaminidase. Although enzymatic characterization of the β-*N*-acetylglucosaminidase produced by *Arenibacter* strains has not been performed yet, there is a possibility to consider these enzymes as good tools for producing biologically active β-*N*-acetylglucosamine derivatives in the near future. α-*N*-Acetylgalactosaminidase of the *Arenibacter* *latericius* is classified as GH109 enzyme. This enzyme exhibits unusual structure and mechanism of action [25,36]. We hypothesize that many of these features may facilitate the beneficial adaptation plasticity of microorganisms in a constantly changing environment of an intertidal flat zone [1,4,5]. A high phylogenetically diversity and broad distribution in marine environments of members of the genus *Arenibacter*, in combination with their various glycoside hydrolase activities, have led to the notion that these microorganisms are specialists for degradation of natural carbohydrates. Moreover, the ability to produce multifarious glycosidases together with the absence of antimicrobial activities suggests that the enzymes play a major role in survival and successful competition of arenibacters in marine microbial communities. In summary, we have demonstrated that taxonomic diverse bacteria of the genus *Arenibacter* has a great potential in production of a wide spectrum of unique glycosidases, which are of special interest in biotechnology and medicine applications.
